# Modest sex differences in the test of basic auditory capabilities (TBAC)

**DOI:** 10.3389/fpsyg.2024.1435529

**Published:** 2024-12-02

**Authors:** Dennis McFadden, Edward G. Pasanen, Gary R. Kidd, Brian Gygi

**Affiliations:** ^1^Department of Psychology, University of Texas at Austin, Austin, TX, United States; ^2^Center for Perceptual Systems, University of Texas at Austin, Austin, TX, United States; ^3^Department of Speech, Language and Hearing Sciences, Indiana University, Bloomington, IN, United States

**Keywords:** auditory ability, individual differences, sex differences, auditory tests, resampling, TBAC

## Abstract

The Test of Basic Auditory Capabilities (TBAC) consists of 19 discrimination and identification tasks selected to study individual differences in audition. In one TBAC study, performance was measured for 340 normal-hearing subjects, but no investigation into possible sex differences was undertaken. That dataset now has been re-analyzed by sex. An effect size for sex difference was calculated for each subtest, and a resampling technique was used to estimate an implied significance for each of those effect sizes. Because almost all the differences observed were small, only the basic outcomes are described here, with more detail provided in Supplementary material. Peripheral physiological measures such as otoacoustic emissions exhibit larger auditory sex differences than do auditory behavioral measures, revealing that those peripheral physiological differences do not propagate simply up the auditory chain.

## 1 Introduction

Although individual differences long have been observed in auditory measures, they never have become a mainstream topic of research. Even so, about 80 years ago, a test for measuring individual differences in musical abilities was developed by Seashore et al. (1956) and, more recently, the Test of Basic Auditory Capabilities (TBAC) was developed (Watson et al., 1976, 1982a, 1982b, 1996; Johnson et al., 1987; Surprenant and Watson, 2002; Kidd et al., 2007).

Individual differences can cluster to produce a group difference, such as a sex difference. Like individual differences, sex differences long have been evident in various physiological and behavioral measures of audition (reviewed in McFadden, 1998; McFadden et al., 2018a, 2018b, 2021), but sex differences also never has become a mainstream topic for auditory research.

The version of the TBAC with the largest set of measures (Kidd et al., 2007) consisted of 19 auditory discrimination and identification subtests that used tones, noise bands, speech, and environmental sounds, with all but one of the subtests using forced-choice tasks. The details of the 19 subtests were provided by Kidd et al. (2007), who used that version of the TBAC to measure individual differences in 340 listeners. Their primary interest was in identifying fundamental auditory abilities underlying the performance on the various subtests. Kidd et al. (2007) mentioned no examination of their dataset for possible sex differences. The large Ns and the large number of tasks studied suggested that basic knowledge about psychoacoustical sex differences could be expanded using that dataset. When informed that authors DM and EGP were interested in analyzing his dataset for sex differences, author GRK kindly provided a spreadsheet containing the relevant data. The original Kidd et al. (2007) dataset now is available online (Kidd et al., 2023).

What follows here is a brief summary of the procedures used for assigning a measure of performance to each subject for each of the 19 subtests, for partitioning the subjects by sex, and for examining the resulting sex differences for each subtest. Greater detail and discussion is provided in Supplementary material (McFadden et al., 2024). One reason for this reporting strategy is that the majority of the sex differences in the Kidd et al. dataset were small and non-significant, making it difficult to justify undue space in an archival journal. On the other hand, the recent movement toward “open science” (Open Science Collaboration, 2015; Nosek et al., 2015) argues that these results do need to be reported (as do failures to replicate) in order for the scientific literature to contain an accurate representation of reality. The twin objectives of preserving journal space while making all legitimate results available archivally are admirably served by the Brief Research Reports published by THIS JOURNAL, in conjunction with Supplementary material containing additional details.

The current results are in accord with an apparent trend in the literature on sex differences in the auditory system: physiological auditory sex differences generally appear to be larger than behavioral auditory sex differences (e.g., McFadden et al., 2018a, 2018b, 2021).

## 2 Methods

All aspects of the Kidd et al. (2007) study were approved by the Institutional Review Board (IRB) of Indiana University, Bloomington, Indiana. No separate IRB approval was required for the analyses reported here because the subjects were identified only by a code number.

### 2.1 Subjects

The subjects were primarily university students who were paid for their services. They ranged in age from 18 to 31 (mean = 22.4 years). Subjects were employed only if they were categorized as “normal hearing” following a standard audiometric screening. Kidd et al. (2007) collected data for 340 subjects and reported on 338 subjects (239 female, 99 male); two subjects were excluded entirely because of poor performance on one or more subtests. In accord with common practice at the time, subjects were offered only the two traditional categories of Female and Male when self-identifying by sex.

### 2.2 Experimental procedures

Groups of up to 12 subjects were tested simultaneously in a large sound-treated room. Subjects were tested for 90 min on each of four consecutive weekdays. Listening was diotic using EAR 3A insert earphones (Etymotic Research, Elk Grove, IL). The standard stimuli for all subtests were presented at 75 dB SPL. The stimulus details for each subtest were provided by Kidd et al. (2007). About 4 years were required to test all 340 subjects.

For 14 of the 19 subtests, the basic procedure was multi-interval forced-choice. There were three observation intervals per trial, with the first interval always containing an example of the standard sound, and the final two intervals containing the standard sound and an alternative sound in random order. The subject’s task always was to identify the observation interval containing the sound *different* from the standard presented in the first interval. For these 14 subtests, trials were presented in blocks of 72, which were organized as 12 groups of 6 trials each. For each group of 6 consecutive trials, the level of difficulty was increased trial-by-trial irrespective of the subject’s response. After the first 36 trials (of 72 trials total), the two easiest stimulus levels were discarded and two harder levels were added. Thus, over the course of 72 trials, eight stimulus levels were presented, and subjects responded on 12 data trials for each of the middle four stimulus levels and responded on 6 data trials for each of the two strongest and the two weakest stimulus levels. No trial-by-trial feedback was given, and chance performance was 50% correct for these 14 tasks.

For 4 of the 19 subtests (Syllable Identification, Nonsense Syllable Identification, Word Identification, Environmental Sounds), there was a single stimulus presentation and the subject selected a response from three or four alternatives presented visually on a computer monitor. Targets were masked by broadband noise, and the signal-to-noise ratio was varied systematically across trials. No trial-by-trial feedback was given, and chance performance was either 33% or 25% correct for different subtests.

The exception to the above forced-choice procedures was Sentence-Identification (subtest 18), which was open set. On each trial, there was a single presentation of a sentence having a length of four to 10 words and masked by broadband noise. The signal-to-noise ratio of the sentences was varied systematically across trials. Each trial had a 6-s response interval, during which the subject wrote the words heard, and an *overall* percentage of correctly identified words was calculated across the 80 trials of the block of trials.

The training prior to data collection was minimal because the goal was to measure existing individual differences relatively quickly, not differences after extensive training (compare Little et al., 2017; McFadden et al., 2018a). Specifically, for each subtest, subjects listened to two example trials using the easiest stimulus level; the correct response was indicated at the end of each example.

### 2.3 Analyses for sex differences

For each subject for each of the subtests, Kidd et al. (2007) calculated an *overall* value of percent correct across all trials (ignoring the differing levels of difficulty of those trials). For each subtest separately, subjects were assigned to one of ten decile groups on the basis of that overall percent correct score; that is, ~34 subjects per decile group. The values of overall percent correct thus calculated for each subtest were the basic measure used for the various analyses reported by Kidd et al. (2007).

Kidd et al. (2007) also calculated other measures of performance, which proved important for the analyses reported here. Specifically, for each subject for each subtest, values of percent correct were calculated for each of the (typically 6–8) levels of difficulty of the task. Then, within each decile group for each subtest, *all* the individual data were fitted with a single sigmoid function, and the stimulus value corresponding to 70% correct decisions was determined. In their Table 3, Kidd et al. (2007) provided those estimated stimulus levels for 70% correct for each of the 10 decile groups for each subtest of the TBAC; those estimates (here called *stimulus values*) permitted the re-analyses for sex differences we are reporting here. Those estimates allowed us to examine sex differences in units of stimulus magnitude, not differences in percent correct.

Our re-analyses began by again using the overall percent correct scores to partition the individual subjects into deciles of ~34 subjects each (sexes pooled) for each of the 19 subtests (To say what surely is obvious: Individual subjects typically fell into different decile groups for different subtests; decile assignment depended solely upon their overall percent correct score across stimulus levels for *that* subtest). Then for each subtest, every subject in each decile group was assigned the *stimulus value* estimated by Kidd et al. (2007) for that decile group (and shown in their Table 3). That is, for each subtest, one group of 34 subjects all were assigned one value of estimated stimulus value, another group of 34 all were assigned another estimated stimulus value, and so on for 10 groups of ~34 subjects each. Only then were subjects sorted by sex. (Note that one consequence of this procedure for assigning stimulus values to individual subjects was that the distributions for the two sex groups likely had smaller variances than would have been the case had separate sigmoid functions been fitted to the data for each subject individually for each subtest.) Smaller stimulus values correspond to better performance for all subtests except for subtest 8 (Syllable Identification), for which the percentage of correctly identified syllables was the measure of performance. In three instances here, a subject having an extremely low percent correct score for an individual subtest was excluded from that analysis (only); Kidd et al. (2007) excluded those subjects from all analyses.

For each sex for each subtest, means, standard deviations, and standard errors were calculated across the assigned estimated stimulus values, and effect sizes were calculated for each sex difference comparison. The effect sizes calculated in this way are called the *actually obtained effect sizes.* Here effect size was calculated as the mean stimulus value for the females minus the mean stimulus value for the males divided by the square root of the weighted mean of the variances of the two distributions (after Cohen, 1992). Effect sizes of 0.2, 0.5, and 0.8 are commonly interpreted as small, medium, and large differences, respectively (see Cohen, 1992).

In order to obtain a perspective on the magnitudes of the various effect sizes actually obtained, a resampling technique was used (see McFadden et al., 2012). Specifically, (1) for each of the 19 subtests separately, the estimated stimulus values for all the female and all the male subjects were pooled into a single group; (2) a random sample the size of the male *N* was identified, and the data for those subjects were extracted for each subtest and designated the “male” group for that resample; (3) the data for the remaining subjects were pooled and designated the “female” group for that resample; (4) using the “male” and “female” groups so formed, an effect size for sex difference was calculated for each subtest based on the estimated stimulus values; (5) those calculated effect sizes were stored; and (6) this resampling process was repeated 20,000 times. Then, (7) for each subtest separately, a tally was done of the number of times an individual resample produced a (n absolute) value of effect size that exceeded the (absolute value of the) actually obtained effect size; (8) that tally was divided by 20,000; and (9) the result was taken as the *implied significance* of the actually obtained effect size for that subtest. Because the tallies were calculated using the *absolute values* of the resampled effect sizes, our estimates of implied significance are “two-tailed” (conservative). Here we use the term “negligible” to denote effect sizes that did not achieve implied significance values of 0.10 (traditionally called marginally significant) or smaller.

## 3 Results

This re-analysis of the Kidd et al. (2007) data uncovered no large sex differences for any of the TBAC subtests. Twelve of the 19 subtests exhibited effect sizes for sex difference smaller than +/−0.20, and the largest effect size was only 0.32. Of the seven subtests whose effect size was greater than 0.20, resampling revealed that only two reached a traditionally accepted level of significance.

The summary statistics are presented in Table 1. The subtest numbers and descriptors (columns 1 and 2) were taken directly from Kidd et al. (2007). Columns 3 and 4 contain the means for the assigned estimated stimulus values for females and males, respectively, and columns 5 and 6 contain the corresponding standard deviations. The effect sizes for each sex difference (females minus males in the numerator) are shown in column 7 of Table 1. The levels of implied significance from resampling are indicated by the superscripts. For most of the subtests, a positive effect size means that males needed a weaker signal than females for 70% correct decisions (males “better” than females); for subtest 8 (Syllable Identification), a *negative* effect size means males were “better.” [Note: Differences are not deficiencies (McFadden et al., 2024)].

**Table 1:** Means, standard deviations (SD), effect sizes (female^a^ minus male^a^) and implied significance levels (superscripts) for all 19 subtests of the TBAC when ALL subjects were included.

(1)	(2)	(3)	(4)	(5)	6	(7)
Subtest Number^b^	Subtest Name and (Units of Measure)^b^	Mean		Standard Deviation		Effect
		Females	Males	Females	Males	Size (3–4)
1	Pitch (ΔF in Hz)	12.68	10.55	9.48	7.20	**0.240**^**f**^
2	Intensity (ΔI in dB)	0.78	0.80	0.57	0.60	−0.044
3	Duration (ΔT in ms)	28.48	25.27	16.16	14.35	**0.205**^**e**^
4	Pulse train (ΔT in ms)	10.24	9.06	5.43	5.21	**0.218**^**e**^
5	Embedded tone (T in ms)	30.09	26.03	12.68	12.95	**0.318**^**g**^
6	Temporal order tones (T in ms)	56.01	50.57	27.73	25.02	**0.201**^**e**^
7	Temp. order syllables (T in ms)	116.56	128.25	48.70	58.11	**−0.226**^**e**^
8^c^	Syllable identification (% Correct)	0.74	0.75	0.06	0.06	−0.126
9	SAM^d^ 8 Hz (mod. depth in dB)	−24.57	−24.55	3.81	3.74	−0.007
10	SAM 20 Hz (dB)	−23.12	−24.02	4.83	4.90	0.185
11	SAM 60 Hz (dB)	−21.21	−21.02	3.55	3.52	−0.056
12	SAM 200 Hz (dB)	−16.83	−17.32	4.05	3.39	0.126
13	Ripple noise (dB)	−5.40	−5.97	3.38	2.95	0.175
14	Gap detection (T in ms)	2.20	2.05	1.23	1.05	0.133
15	Gap discrimination (ΔT in ms)	38.00	35.10	14.34	13.61	**0.205**^**e**^
16^c^	Syllable (CVC) identif. (S/N)	−7.54	−7.59	1.53	1.52	0.029
17^c^	Word identification (S/N)	−10.50	−10.59	1.48	1.77	0.054
18^c^	Sentence identification (S/N)	−8.24	−8.21	0.56	0.60	−0.047
19^c^	Environmental sound identif. (S/N)	−12.99	−13.17	1.13	1.06	0.163

a Female *N* = 240 for most subtests; Male *N* = 100 for most subtests; otherwise 239 or 99, respectively.

b From Kidd et al. (2007).

c Subtests employing single stimulus presentations; all others were 3-interval, 2-alternative forced choice.

d Sinusoidal amplitude modulation of a noise band.

e Implied significance, from resampling: 0.05 < *p* < 0.10.

f Implied significance, from resampling: 0.01 < *p* < 0.05.

g Implied significance, from resampling: 0.001 < *p* < 0.01.

In column 7 of Table 1, bold font is used to indicate the seven TBAC subtests having effect sizes for sex difference greater than 0.2 (absolute values); note that five of those seven differences were only marginally significant under resampling. The largest sex difference was for the TBAC subtest Kidd et al. (2007) called Embedded Tone (effect size ≅ 0.32). That task required detecting the presence/absence of a brief tone in the middle of a sequence of nine brief tones of random frequency. That is, it was a rather complex task compared to most psychoacoustical tasks. The TBAC subtest with the second largest sex difference was called Pitch (subtest 1 in Table 1; effect size ≅ 0.24). This was a traditional frequency-discrimination task; the standard tone was 1.0 kHz and 250 ms in duration, and the frequency of the comparison tone varied from 1.002 to 1.256 kHz on different trials. Thus, this task involved considerably less-complex stimuli than the Embedded Tone task. Like Kidd et al. (2007), Rammsayer and Troche (2012) also observed males performing better than females at a frequency-discrimination task using pure tones (effect size = 0.62).

The remainder of Table 1 speaks for itself. Twelve of the subtests had sex differences that were negligible and not significant according to resampling. Those subtests (not flagged) for which one female or one male was excluded because of extreme performance (subtests 13, 14, and 18) exhibited negligible effect sizes, and N was not a contributing factor.

Kidd et al. (2007, their Table 2) reported split-half reliability coefficients for all 19 subtests. Of those seven subtests exhibiting an effect size for sex difference greater than 0.2 here (Table 1), all but one had a reliability coefficient greater than 0.72; the largest coefficient was 0.82 (Pitch and Pulse Train) and the smallest was 0.61 (Gap Discrimination).

Kidd et al. (2007) performed a factor analysis of their TBAC results. For that analysis, they used arcsine-transformed values of percent correct scores, a measure that did not constrain extreme values (unlike our estimated stimulus values). The result was four orthogonal factors that accounted for approximately 50% of the variance across the 19 subtests. The two subtests showing the largest sex differences in the current re-analysis (Embedded Tone and Pitch), as well as subtests 7 and 15, all loaded highly on the same factor, called Pitch and Time. The other subtests in Table 1 that exhibited marginal significance loaded highly on the factor called Loudness and Duration. No sex differences were found for any of the subtests that loaded highly on the remaining factors called Amplitude Modulation and Familiar Sounds. That pattern of results suggests that future investigators hoping to identify the underpinnings of sex differences in auditory behavior should focus on subtests involving Pitch and Time. Kidd et al. (2007), especially their Figure 2, provides more information about the factor structure underlying the TBAC results.

### 3.1 Race/ethnicity

Individual differences also can cluster to produce apparent race/ethnicity differences (reviewed by McFadden et al., 2018a, 2018b, 2021). Estimating race differences in the Kidd et al. (2007) dataset is difficult because of the small number of non-White subjects. The topic is discussed in the Supplementary material (McFadden et al., 2024).

## 4 Discussion

When the 19 subtests of the TBAC were examined for sex differences, the majority of the effect sizes were negligible and not significant according to resampling (see Table 1). Only one subtest exhibited an effect size greater than 0.3 (Embedded Tone). Even though that outcome was highly significant, effect sizes between 0.2 and 0.5 are viewed as only small-to-medium effects (Cohen, 1992). Additional detail and discussion can be found in McFadden et al. (2024).

The auditory literature contains numerous reports of sex differences in behavioral measures (reviewed by McFadden, 1998, and McFadden et al., 2018a). Those reports suggest that females have better hearing sensitivity than males, exhibit less temporary and permanent noise-induced hearing loss than males, have more overshoot than males (stronger cochlear amplifiers?), and have less two-tone suppression than males. By comparison, males appear to be more sensitive to both interaural time and interaural level differences (McFadden, 1998, Figure 1), more sensitive to the cubic difference tone generated between some tonal signals and tonal maskers (McFadden et al., 2012, 2018a), and more sensitive in the Neff et al. (1996) simultaneous-masking task. To the latter list now can be added better male performance in the Embedded Tone and Pitch subtests of the TBAC.

The literature also contains numerous reports of sex differences in physiological measures of the auditory periphery such as otoacoustic emissions (OAEs) and click-evoked auditory evoked potentials (AEPs) (reviewed by McFadden, 1998, and McFadden et al., 2018b, 2021). In general, the sex differences for OAEs and AEPs have been larger than the sex differences reported for auditory behavior. One reason these physiological sex differences are interesting is that they are correlated with subject variables such as twinship, sexual orientation, heritability, and degree of exposure to androgens prenatally (reviewed by McFadden, 2002, 2008, 2009, 2011; McFadden and Champlin, 2000). Also interesting is the generally weak correlations between the physiological measures and the psychoacoustical measures (McFadden et al., 2012, 2018b, 2021). The generally larger sex differences for physiological measures than behavioral measures reveals that the peripheral differences do not propagate simply up the auditory chain. The implication is that physiological sex differences likely will provide more insights into underlying auditory mechanisms than will psychoacoustical sex differences.

Although no large sex differences were revealed in the TBAC subtests, large differences have been reported for other auditory behavioral tasks (Neff et al., 1996; summary in McFadden et al., 2012, 2018a) and for physiological auditory measures such as OAEs and AEPs (summary in McFadden et al., 2012, 2018b, 2021). It is tempting to ponder the possible origins of those larger sex differences. Considerable evidence suggests that the sex differences for OAEs are largely attributable to differential exposure to androgens during prenatal development (McFadden, 2002, 2008, 2009, 2011). The emergence of sex differences in click-evoked AEPs just prior to puberty (e.g., Krizman et al., 2019) also suggests the involvement of sex hormones. Understanding behavioral auditory sex differences is more tricky because they could be solely physiological, solely due to differential experience, or some combination of those two. A recent multi-year study examined possible correlations between various OAE and AEP measures with performance in seven different auditory behavioral tasks (McFadden et al., 2018a, 2018b, 2021). That is, might individual (and sex) differences in behavior be explained by individual differences in certain peripheral physiological measures? The results were underwhelming. Few strong correlations between behavior and physiology were observed (compare Walsh et al., 2010). Accordingly, we hope the reader will understand our reluctance to speculate about possible bases for the small sex differences observed in the TBAC subtests.

As personalized medicine becomes more established as a goal in clinical practice and medical research (e.g., Goetz and Schork, 2018), individual differences of various sorts will become of increasing interest. Clusters of individual differences such as sex and race/ethnic differences also will be of increasing interest. Individual, sex, and/or race differences in the auditory system may prove to have predictive value for prevention or treatment for maladies of the auditory system or for correlated maladies in other systems or organs. The small sizes of the current sex differences do not preclude larger, perhaps clinically relevant, differences for other auditory measures, particularly physiological measures.

It is important to recognize that essentially the entire corpus of current knowledge about human hearing, both physiological and psychoacoustical measures, comes from research done at universities in north America and western Europe and thus is based almost exclusively on White subjects. The existence, and size, of sex (and race) differences in other cultural groups still is unknown, meaning that clinically relevant differences may exist in those groups.

The small sizes of the behavioral differences reported here suggest that they are likely to arise as incidental by-products of responses to evolutionary pressures on characteristics other than audition.

## Data Availability

The original data on which the present analyses were based can be found in Kidd et al., 2023. Further inquiries can be directed to author G. R. Kidd.

## Author’s note

Dr. Charles S. Watson was a student of individual differences in audition, and he was instrumental in obtaining the funding for, implementing, analyzing, writing, and publishing the original study on which this re-analysis is based (Kidd et al., 2007). Because Dr. Watson died prior to the beginning of this re-analysis, the policies of this journal prevent him from being listed as a co-author here even though his contributions were fundamental.

## References

[ref1] Cohen J. (1992). A power primer. Psychol. Bull. 112, 155–159. doi: 10.1037/0033-2909.112.1.155, PMID: 19565683

[ref2] Goetz L. H. Schork N. J. (2018). Personalized medicine: motivation, challenges, and progress. Fertil. Steril. 109, 952–963. doi: 10.1016/j.fertnstert.2018.05.006, PMID: 29935653 PMC6366451

[ref3] Johnson D. M. Watson C. S. Jensen J. K. (1987). Individual differences in auditory capabilities. J. Acoust. Soc. Am. 81, 427–438. doi: 10.1121/1.394907, PMID: 3558959

[ref4] Kidd G. R. Watson C. S. Gygi B. (2007). Individual differences in auditory abilities. J. Acoust. Soc. Am. 122, 418–435. doi: 10.1121/1.2743154 17614500

[ref5] Kidd G. R. Watson C. S. Gygi B. (2023). Individual differences in auditory abilities: the expanded TBAC dataset. Indiana University DataCORE. doi: 10.5967/wv6d-n347 17614500

[ref6] Krizman J. Bonacina S. Kraus N. (2019). Sex differences in subcortical auditory processing emerge across development. Hear. Res. 380, 166–174. doi: 10.1016/j.heares.2019.07.002, PMID: 31306931

[ref7] Little D. F. Zhang Y.-X. Wright B. A. (2017). Disruption of perceptual learning by a brief practice break. Curr. Biol. 27, 3699–3705.e3. doi: 10.1016/j.cub.2017.10.032, PMID: 29174894 PMC5848209

[ref8] McFadden D. (1998). Sex differences in the auditory system. Devel. Neuropsych. 14, 261–298. doi: 10.1080/87565649809540712

[ref9] McFadden D. (2002). Masculinization effects in the auditory system. Arch. Sex. Behav. 31, 99–111. doi: 10.1023/A:1014087319682 11910797

[ref10] McFadden D. (2008). What do sex, twins, spotted hyenas, ADHD, and sexual orientation have in common? Perspect. Psychol. Sci. 3, 309–323. doi: 10.1111/j.1745-6924.2008.00082.x, PMID: 26158951

[ref11] McFadden D. (2009). Masculinization of the mammalian cochlea. Hear. Res. 252, 37–48. doi: 10.1016/j.heares.2009.01.002, PMID: 19272340 PMC2698035

[ref12] McFadden D. (2011). Sexual orientation and the auditory system. Front. Neuroendocrinol. 32, 201–213. doi: 10.1016/j.yfrne.2011.02.001, PMID: 21310172 PMC3085661

[ref13] McFadden D. Champlin C. A. (2000). Comparison of auditory evoked potentials in heterosexual, homosexual, and bisexual males and females. J. Assoc. Res. Otolaryngol. 1, 89–99. doi: 10.1007/s101620010008, PMID: 11548240 PMC2504562

[ref14] McFadden D. Champlin C. A. Pho M. H. Pasanen E. G. Maloney M. M. Leshikar E. M. (2021). Auditory evoked potentials: differences by sex, race, and menstrual cycle and correlations with common psychoacoustical tasks. PLOS ONE 16:e0251363. doi: 10.1371/journal.pone.0251363, PMID: 33979393 PMC8115856

[ref15] McFadden D. Pasanen E. G. Leshikar E. M. Hsieh M. D. Maloney M. M. (2012). Comparing behavioral and physiological measures of combination tones: sex and race differences. J. Acoust. Soc. Am. 132, 968–983. doi: 10.1121/1.4731224, PMID: 22894218 PMC3427363

[ref16] McFadden D. Pasanen E. G. Maloney M. M. Leshikar E. M. Pho M. H. (2018a). Differences in common psychoacoustical tasks by sex, menstrual cycle, and race. J. Acoust. Soc. Am. 143, 2338–2354. doi: 10.1121/1.5030998, PMID: 29716303 PMC5915329

[ref17] McFadden D. Pasanen E. G. Maloney M. M. Leshikar E. M. Pho M. H. (2018b). Correlations between otoacoustic emissions and performance in common psychoacoustical tasks. J. Acoust. Soc. Am. 143, 2355–2367. doi: 10.1121/1.5030999, PMID: 29716248 PMC5915325

[ref18] McFadden D. Pasanen E. P. Kidd G. R. Watson C. S. Gygi B. (2024). Supplement to modest sex differences in the test of basic auditory capabilities (TBAC). Texas ScholarWorks. doi: 10.26153/tsw/44104 PMC11647213 39687566

[ref19] Neff D. L. Kessler C. J. Dethlefs T. M. (1996). Sex differences in simultaneous masking with random-frequency maskers. J. Acoust. Soc. Am. 100, 2547–2550. doi: 10.1121/1.417364 8865658

[ref20] Nosek B. A. Alter G. Banks G. C. Borsboom D. Bowman S. D. Breckler S. J.. (2015). Promoting an open research culture. Science 348, 1422–1425. doi: 10.1126/science.aab2374, PMID: 26113702 PMC4550299

[ref21] Open Science Collaboration (2015). Estimating the reproducibility of psychological science. Science 349:aac4716. doi: 10.1126/science.aac4716 26315443

[ref22] Rammsayer T. H. Troche S. J. (2012). On sex-related differences in auditory and visual sensory functioning. Arch. Sex. Behav. 41, 583–590. doi: 10.1007/s10508-011-9880-8, PMID: 22183583

[ref001] Seashore C. E. Lewis D. Saetveit J. G. (1956). Seashore measures of musical talents. New York: Psychological Corp.

[ref23] Surprenant A. M. Watson C. S. (2002). Individual differences in the processing of speech and nonspeech sounds by normal-hearing listeners. J. Acoust. Soc. Am. 110, 2085–2095. doi: 10.1121/1.1404973 11681386

[ref002] Walsh K. P. Pasanen E. G. McFadden D. (2010). Overshoot measured physiologically and psychophysically in the same human ears. Hear. Res. 268, 22–37. 20430072 10.1016/j.heares.2010.04.007 PMC2923227

[ref24] Watson C. S. Jensen J. K. Foyle D. C. Leek M. R. Goldgar D. E. (1982a). Performance of 146 normal adult listeners on a battery of auditory discrimination tasks. J. Acoust. Soc. Am. 71:S73. doi: 10.1121/1.2019533

[ref25] Watson C. S. Johnson D. M. Lehman J. R. Kelly W. J. Jensen J. K. (1982b). An auditory discrimination test battery. J. Acoust. Soc. Am. 71:S73. doi: 10.1121/1.2019532

[ref26] Watson C. S. Kelly W. J. Wroton H. W. (1976). Factors in the discrimination of tonal patterns. II. Selective attention and learning under various levels of stimulus uncertainty. J. Acoust. Soc. Am. 60, 1176–1186. doi: 10.1121/1.381220, PMID: 977844

[ref27] Watson C. S. Qui W. W. Chamberlain M. Li X. (1996). Auditory and visual speech perception: confirmation of a modality-independent source of individual differences in speech recognition. J. Acoust. Soc. Am. 100, 1153–1162. doi: 10.1121/1.416300, PMID: 8759968

