# Supplementary material for: Modest sex differences in the test of basic auditory capabilities (TBAC)
Source: Front Psychol. 2024 Dec 2;15:1435529. doi: 10.3389/fpsyg.2024.1435529 (PMC11647213; doi:10.3389/fpsyg.2024.1435529)
Supplement: Supplementary file 1 [file Data_Sheet_1.pdf]

This is an **Online Supplement** (http://dx.doi.org/10.26153/tsw/44104) to a published article entitled Modest sex differences in the Test of Basic Auditory Capabilities (TBAC), Frontiers in Psychology/Auditory Cognitive Neuroscience 15:1435529.
doi: 10.3389/fpsyg.2024.1435529
by D. McFadden, E.G. Pasanen, G.R. Kidd, and B. Gygi,

NOTE: The organization here is parallel to that in the published version, but considerably more detail is provided here. In order to tell a coherent story, some of the text here duplicates text published in McFadden et al. (2024*).
= =

Online Supplement to

Modest Sex Differences in the Test of Basic Auditory Capabilities (TBAC)

Dennis McFadden[1*], Edward G. Pasanen[1], Gary R. Kidd[2], and Brian Gygi[2]

[1]Department of Psychology and Center for Perceptual Systems, University of Texas,

108 East Dean Keeton, A8000, Austin, Texas, USA 78712-1043

[2]Department of Speech, Language, and Hearing Sciences, Indiana University,

IU Health Sciences Building, 2631 East Discovery Parkway, Bloomington, Indiana, USA 47408

[*] **Correspondence**:

Dennis McFadden

mcfadden@utexas.edu

Key Words: auditory ability; individual differences; sex difference; auditory tests; resampling;

TBAC

ABSTRACT

Kidd et al. (2007) measured performance on 19 auditory discrimination and identification tasks for 338 normal-hearing subjects. No examination of possible sex differences was reported. That dataset was re-analyzed for sex differences; a brief account of the results was published (McFadden et al., 2024**), and full results are reported here. An effect size for sex difference was calculated for each subtest, and a resampling technique was used to estimate an implied significance for each of those effect sizes. Two of the 19 auditory tasks did exhibit significant sex differences, and five more were marginally significant, but all the effect sizes were small (<0.32). The largest sex difference was for an unusual task requiring the subject to detect the presence/absence of a brief tone in the middle of a sequence of nine brief tones of random frequency. The second largest sex difference was for a traditional frequency-discrimination task, replicating a previous finding. Because all sex differences observed were small, they are of interest primarily to basic science, not clinical practice. The emerging picture is that the marked sex differences seen in peripheral physiological measures do not propagate simply through the auditory chain into psychoacoustical differences. Possible race/ethnic differences were examined but because only 10% of the TBAC subjects self-identified as Non-White, those results are only suggestive.

Key Words: auditory ability; individual differences; sex difference; race difference

Isupp. INTRODUCTION

Pronounced individual differences long have been observed in both physiological and behavioral measures of hearing, but they never have been a mainstream topic in auditory research. One research group that did make considerable contributions to the study of individual differences consisted of C.S. Watson and his colleagues, who developed a Test of Basic Auditory Capabilities (TBAC) that they administered to hundreds of subjects over the years (Watson et al., 1976, 1982a, 1982b, 1996; Johnson et al., 1987; Surprenant and Watson, 2001; Kidd et al, 2007). The TBAC eventually consisted of 19 subtests measuring discrimination and identification using tones, noise bands, speech, and environmental sounds, all but one subtest using forced-choice tasks (see Kidd et al., 2007). The current version of the TBAC is commercially available from Communication Disorders Technology, Inc. (Bloomington, IN) at http://www.comdistec.com/new/TBAC.html.

Individual differences can cluster to produce a group difference, such as a sex difference. Sex differences long have been evident in various physiological and behavioral measures of audition (reviewed by McFadden, 1998; McFadden et al., 2018a, 2018b, 2021), but like individual differences, sex differences also never has emerged as a mainstream area of auditory research. Three recent articles have reported sex differences for otoacoustic emissions (OAEs), auditory evoked potentials (AEPs), and seven common psychoacoustical tasks (McFadden et al, 2018b, 2021, and 2018a, respectively). One primary finding was that (in terms of effect size), the largest of the sex differences for the physiological measures were larger than the largest of the sex differences for the behavioral measures. Second, those physiological measures showing sex differences were stronger ("better") for the females than for the males. Third, the correlations between physiological and behavioral measures typically were weak; that is, the existence of substantial physiological sex differences at the auditory periphery did not produce obligatory widespread sex differences in behavior. Taken together, this evidence suggests that physiological sex differences likely will provide more insights into underlying auditory mechanisms than will psychoacoustical sex differences.

The sex differences in OAEs are interesting because they exist at birth (Strickland et al. 1985; Burns et al., 1992), apparently remain reasonably constant through life (Burns, 2017), and are correlated with sexual orientation (McFadden and Pasanen, 1998, 1999). This suggests that a common prenatal hormonal mechanism or event may underlie all of these findings (McFadden, 2002, 2008, 2009, 2011). AEPs exhibit sex differences beginning about puberty (Krizman et al., 2019), and they also correlate with sexual orientation later in life (McFadden and Champlin, 2000).

Kidd et al. (2007) did not analyze their TBAC data for sex differences. The large Ns and the large number of tasks studied suggested that knowledge about psychoacoustical sex differences could be expanded from that dataset. When Dr. Kidd was asked if his team was willing to allow someone else to analyze their data for sex differences, he graciously agreed and provided the relevant data. The results reported here come exclusively from the Kidd et al. dataset, which is available online (Kidd et al., 2023).

Individual differences also can cluster to produce an apparent race/ethnic difference. The auditory literature contains examples of race/ethnic differences in OAEs, AEPs, hearing sensitivity, susceptibility to temporary and permanent noise-induced hearing loss, and incidence and severity of tinnitus (summarized in McFadden et al., 2018b). In addition, there are suggestions that sex differences can interact with race/ethnic differences (see McFadden et al. 2018a, 2018b, 2021). Because of these findings about possible interactions between sex differences and race/ethnic differences, we also examined the Kidd et al. (2007) dataset for possible race differences. However, 90% of the subjects in that study identified as "White with no Hispanic background" on the two standard items on race/ethnicity required by the National Institutes of Health (reproduced in section IIIsupp. B. below), meaning that the Non-White Ns were too small to provide conclusive evidence about any contribution of race/ethnicity to the TBAC. Accordingly, those analyses were not reported in the published version of this report (McFadden et al., 2024**), but are reported in this online supplement.

Because this report is about sex (and race) differences, there must be no uncertainty about the views of the authors on these matters. **Differences are not**

deficiencies, and no difference reported here should be interpreted as indicating a deficiency. In the end, all of the sex (and race) differences reported here were too small to affect everyday listening. This renders these differences primarily of interest for basic auditory science, not clinical practice.

FOOTNOTE 1 -- As noted elsewhere (e.g., McFadden et al., 2021), we believe that race categories are a poor proxy for what is likely to be one of the actual reasons for some apparent "race" differences in audition: namely, individual differences in pigmentation (because melanocytes exist in the cochlea and appear to be involved in cochlear homeostasis; see McFadden et al., 2018a; McFadden 2011). This melanin connection is most likely to be relevant for those physiological measures that are most dependent upon individual differences in the cochlea. By comparison, any substantial race differences found for psychoacoustical tasks are unlikely to originate solely from differences in cochlear melanocytes.

Even though the sex (and race) differences in the TBAC tasks proved to be small, the recent movement toward "open science" (Open Science Collaboration, 2015; Nosek et al., 2015) argues that those results do need to be reported (as well as failures to replicate) in order for the scientific literature to contain an accurate representation of reality. Hence the strategy of reporting these results by pairing a brief published version (McFadden et al., 2024**) with this more complete online version.

Note again that some of the text here duplicates text in our published report (McFadden et al., 2024**) without quotation marks.

The current version of the TBAC is commercially available from Communication Disorders Technology, Inc. (Bloomington, IN) at http://www.comdistec.com/new/TBAC.html. It consists of nine subtests, not all 19 re-analyzed here. The included subtests are numbers 1-8 of the subtests listed in Table III of Kidd et al. (2007), plus a version of the Environmental Sound Recognition test (subtest 19). The subtests shown here to exhibit the largest sex differences are included in the current version of the TBAC.

IIsupp. METHODS

As noted, the TBAC consists of 19 auditory tasks requiring discrimination and identification and employs tones, noise bands, and speech, and environmental sounds as stimuli. The details of the various tasks were described in Kidd et al. (2007), and some are discussed below. All aspects of the Kidd et al. study were approved by the Institutional Review Board of Indiana University, Bloomington, Indiana. No IRB approval was required for the analyses reported here because the subjects were identified only by a code number.

IIsupp. A. Subjects

The subjects were primarily university students who were paid for their services. They were employed only if they were categorized as "normal hearing" following a standard audiometric screening. Kidd et al. (2007) collected data for 340 subjects and reported on 338 subjects (239 female, 99 male); two subjects were excluded entirely because of poor performance on one or more subtests.

IIsupp. B. Experimental procedures

Groups of up to 12 subjects were tested simultaneously in a large sound-treated room. Subjects were tested for 90 minutes on each of four consecutive weekdays. Listening was diotic using EAR 3A insert earphones (Etymotic Research, Elk Grove, IL). The standard stimuli for all subtests were presented at 75 dB SPL. The stimulus details for each subtest were provided by Kidd et al. (2007).

For 14 of the 19 subtests, the basic procedure was multi-interval forced-choice. There were three observation intervals per trial, with the first interval always containing an example of the standard sound, and the final two intervals containing the standard sound and an alternative sound in random order. The subject's task always was to identify the observation interval containing the sound *different* from the standard presented in the first interval. For these 14 subtests, trials were presented in blocks of 72, which were organized as 12 groups of 6 trials each. For each group of 6 consecutive trials, the level of difficulty was increased trial-by-trial irrespective of the subject's response. After the first 36 trials (of 72 trials total), the two easiest stimulus levels were discarded and two harder levels were added. Thus, over the course of 72

trials, eight stimulus levels were presented, and subjects responded on 12 data trials for each of the middle four stimulus levels and responded on 6 data trials for each of the two easiest and the two hardest stimulus levels. No trial-by-trial feedback was given. Chance performance was 50% correct.

For 4 of the 19 subtests (Syllable Identification, Nonsense Syllable Identification, Word Identification, Environmental Sounds), there was a single stimulus presentation and the subject selected a response from three or four alternatives presented visually on a computer monitor. Targets were masked by broadband noise, and the signal-to-noise ratio was varied systematically across trials. No trial-by-trial feedback was given. Chance performance was either 33% or 25% correct for different subtests.

The exception to the above forced-choice procedures was the Sentence-Identification subtest (#18), which was open set. On each trial, there was a single presentation of a sentence having a length of four to ten words and masked by broadband noise. The signal-to-noise ratio of the sentences was varied systematically across trials. Each trial had a 6-s response interval, during which the subject wrote the words heard, and an *overall* percentage of correctly identified words was calculated across the 80 trials of the block of trials.

For each subtest, subjects listened to two example trials using the easiest stimulus level; the correct response was indicated at the end of each example. The goal was to measure existing individual differences relatively quickly, not differences after extensive training (compare Little et al., 2017; McFadden et al., 2018a).

IIsupp. C. Analyses for sex differences

For each subject for each of the subtests, Kidd et al. (2007) calculated an *overall* value of percent correct across all trials (ignoring the differing levels of difficulty of those trials). For each subtest separately, subjects were assigned to one of ten decile groups on the basis of that overall percent correct score; that is, ~34 subjects per decile group. The values of overall percent correct thus calculated for each subtest were the basic measure used for the various analyses reported by Kidd et al. (2007).

Kidd et al. (2007) also calculated other measures of performance, which proved important for the analyses reported here. Specifically, for each subject for each subtest,

values of percent correct were calculated for each of the (typically 6 - 8) levels of difficulty of the task. Then, within each decile group for each subtest, *all* the individual data were fitted with a single sigmoid function, and the stimulus value corresponding to 70% correct decisions was determined. In their Table III, Kidd et al. (2007) provided those estimated stimulus levels for 70% correct for each of the 10 decile groups for each subtest of the TBAC; those estimates (here called *stimulus values*) permitted the re-analyses for sex differences we are reporting here.

Our re-analysis began by again using the overall percent correct scores to partition the individual subjects into deciles of ~34 subjects each (sexes pooled) for each of the 19 subtests. (To say what surely is obvious: Individual subjects typically fell into different decile groups for different subtests; decile assignment depended solely upon their *overall* percent correct score across stimulus levels for *that* subtest.) Then for each subtest, every subject in each decile group was assigned the stimulus value estimated by Kidd et al. (2007) for that decile group and shown in their Table III. That is, for each subtest, one group of 34 subjects all were assigned one value of estimated stimulus value, another group of 34 all were assigned another estimated stimulus value, and so on for ten groups of ~34 subjects each. Only then were subjects sorted by sex. (Note that one consequence of this procedure for assigning stimulus values to individual subjects was that the distributions for the two sex groups likely had smaller variances than would have been the case had separate sigmoid functions been fitted to the data for each subject individually for each subtest.) In three instances here, a subject having an extremely low percent correct score for an individual subtest was excluded from *that* analysis; Kidd et al. (2007) excluded those subjects from all analyses.

For each sex for each subtest, means, standard deviations, and standard errors were calculated across those assigned estimated stimulus values, and effect sizes were calculated for each sex difference comparison. The effect sizes calculated in this way are called the *actually obtained effect sizes.* Here effect size was calculated as the mean for the females minus the mean for the males divided by the square root of the weighted mean of the variances of the two distributions. Effect sizes of 0.2, 0.5, and 0,8 are commonly interpreted as small, medium, and large differences, respectively (see Cohen, 1992).

In order to obtain a perspective on the magnitudes of the various effect sizes actually obtained, a resampling technique was used (see McFadden et al., 2012). Specifically, (1) for each of the 19 subtests separately, the estimated stimulus values for all the female and all the male subjects were pooled into single groups; (2) a random sample the size of the male N was identified, and the data for those subjects were extracted for each subtest and designated the "male" group; (3) the data for the remaining subjects were pooled and designated the "female" group; (4) using the "male" and "female" groups so formed, an effect size for sex difference was calculated for each subtest based on the estimated stimulus values; (5) those calculated effect sizes were stored; and (6) this resampling process was repeated 20,000 times. Then, (7) for each subtest separately, a tally was done of the number of times a resample produced a(n absolute) value of effect size that exceeded the (absolute value of the) actually obtained effect size; (8) that tally was divided by 20,000; and (9) the result was taken as the *implied significance* of the actually obtained effect size for that subtest. Because the tallies were calculated using the *absolute values* of the resampled effect sizes, our estimates of implied significance are "two-tailed" (conservative). Here we use the term “negligible” to denote effect sizes that did not achieve implied significance values of 0.10 (marginally significant) or smaller.

IIIsupp. RESULTS

IIIsupp. A. All subjects

The effect sizes for sex difference were small for all 19 subtests of the TBAC. The summary statistics and implied significance levels are presented in Table SI (duplicated here from McFadden et al., 2024*, for the convenience of the reader). The subtest numbers and descriptors (columns 1 and 2) were taken directly from Kidd et al. (2007). Columns 3 and 4 contain the means for the assigned estimated stimulus values for females and males, respectively, and columns 5 and 6 contain the corresponding standard deviations. The effect sizes for each sex difference (females minus males in the numerator) are shown in column 7 of Table SI, and those effect sizes greater than 0.2 are shown in **bold** font (by convention, 0.2 is a small effect; see Cohen, 1992). The levels of implied significance from resampling are indicated by the superscripts. For

most of the subtests, a positive effect size means that males needed a weaker signal than females for 70% correct decisions; for subtest 8 (Syllable Identification), a negative effect size means males were "better."

Table SI. Means, standard deviations (SD), effect sizes (female[a] minus male[a]) and implied significance levels (superscripts) for all 19 subtests of the TBAC when ALL subjects were included.						
(1)	(2)	(3)	(4)	(5)	6	(7)
Subtest	Subtest Name	Mean		Standard Deviation		Effect
Number[b]	and (Units of Measure)[b]	Females	Males	Females	Males	Size (3 - 4)
1	Pitch (ΔF in Hz)	12.68	10.55	9.48	7.20	**0.240[f]**
2	Intensity (ΔI in dB)	0.78	0.80	0.57	0.60	-0.044
3	Duration (ΔT in ms)	28.48	25.27	16.16	14.35	**0.205[e]**
4	Pulse train (ΔT in ms)	10.24	9.06	5.43	5.21	**0.218[e]**
5	Embedded tone (T in ms)	30.09	26.03	12.68	12.95	**0.318[g]**
6	Temporal order tones (T in ms)	56.01	50.57	27.73	25.02	**0.201[e]**
7	Temp. order syllables (T in ms)	116.56	128.25	48.70	58.11	**-0.226[e]**
8[c]	Syllable identification (% Correct)	0.74	0.75	0.06	0.06	-0.126
9	SAM[c] 8 Hz (mod. depth in dB)	-24.57	-24.55	3.81	3.74	-0.007
10	SAM 20 Hz (dB)	-23.12	-24.02	4.83	4.90	0.185
11	SAM 60 Hz (dB)	-21.21	-21.02	3.55	3.52	-0.056
12	SAM 200 Hz (dB)	-16.83	-17.32	4.05	3.39	0.126
13	Ripple noise (dB)	-5.40	-5.97	3.38	2.95	0.175
14	Gap detection (T in ms)	2.20	2.05	1.23	1.05	0.133
15	Gap discrimination (ΔT in ms)	38.00	35.10	14.34	13.61	**0.205[e]**
16[c]	Syllable (CVC) identif. (S/N)	-7.54	-7.59	1.53	1.52	0.029
17[c]	Word identification (S/N)	-10.50	-10.59	1.48	1.77	0.054
18[c]	Sentence identification (S/N)	-8.24	-8.21	0.56	0.60	-0.047
19[c]	Environmental sound identif. (S/N)	-12.99	-13.17	1.13	1.06	0.163
[a]Female N = 240 for most subtests; Male N = 100 for most subtests; otherwise 239 or 99, respectively						
[b]From Kidd et al. (2007)						
[c]Subtests employing single stimulus presentations; all others were 3-interval, 2-alternative forced choice						
[d]Sinusoidal amplitude modulation of a noise band						
[e]Implied significance, from resampling: 0.05 < p < 0.10						
[f]Implied significance, from resampling: 0.01 < p < 0.05						
[g]Implied significance, from resampling: 0.001 < p < 0.01						

Column 7 of Table SI reveals that seven of the 19 subtests had effect sizes for sex difference greater than 0.2 (absolute values), but five of those seven differences were only marginally significant under resampling. The largest sex difference was for the TBAC subtest Kidd et al. (2007) called Embedded Tone (effect size ≅ 0.32). This task required detecting the presence/absence of a brief tone in the middle of a sequence of nine brief tones of random frequency (details in section IVsupp). That is, it was rather a complex task compared to most psychoacoustical tasks. The TBAC subtest with the second largest sex difference was called Pitch (subtest 1 in Table SI; effect size ≅ 0.24). This was a traditional frequency-discrimination task; the standard tone was 1.0 kHz and 250 ms in duration, and the frequency of the comparison tone varied from 1.002 to 1.256 kHz on different trials. Thus, this task involved considerably less-complex stimuli than the Embedded Tone task. Like Kidd et al. (2007), Rammsayer and Troche (2012) also observed males performing better than females at a frequency-discrimination task using pure tones (effect size = 0.62). These two tasks are discussed in more detail in Section IVsupp below.

The remainder of Table SI speaks for itself. Twelve of the subtests had sex differences that were negligible and not significant. Those subtests (not flagged) for which one female or one male was excluded because of extreme performance (subtests 13, 14, and 18) exhibited negligible effect sizes, and N was not a contributing factor.

IIIsupp. B. Race as a subject variable

The Kidd et al. (2007) study required each subject to self-categorize on race/ethnicity by answering the two standard items required by the National Institutes of Health. Namely,

Item 1. (Yes or No) Are you of Hispanic, Latino, or Spanish Origin (Cuban, Mexican, Puerto Rican, South or Central American, or other Spanish culture or origin, regardless of race)?

Item 2. (Pick One) Are you American Indian or Alaskan native; Asian; Black or African American; Native Hawaiian or Other Pacific Islander; White; More than one race; Other or Unknown (please specify if known).

Because of the various past findings about race/ethnic effects on auditory measures (e.g., McFadden et al., 2018a, 2018b, 2021), a logical strategy for this re-analysis would have been to partition these subjects into several different race/ethnic groups, calculate the effect sizes for sex difference for those different groups, and then repeat the resampling procedures described above for each race/ethnic group separately. That strategy was rejected, however, because out of the total of 340 subjects, only 27 females and 7 males self-identified with any race/ethnicity category other than "White, not of Hispanic origin." Those residual Ns clearly were inadequate to provide convincing conclusions, so all analyses reported in McFadden et al. (2024**) and in Table SI above included ALL subjects, regardless of their self-identifications by race/ethnicity on the two NIH items.

Even though the number of subjects in the Kidd et al. (2007) study who self-identified as Non-White was too small for separate analyses for sex differences, it was possible to take a half-step in that direction. Specifically, in the spirit of the open-science initiative (Open Science Collaboration, 2015; Nosek et al., 2015), we calculated means, standard deviations, and effect sizes only for those subjects who self-identified as White. Those summary statistics are shown for each of the 19 subtests in Table S2. Because the number of Non-White subjects was so small compared to the number of White subjects, there should be little surprise that the pattern of results for Whites Only (Table S2) is highly similar to the pattern when ALL subjects were included (Table SI). The largest effect size again was for the Embedded Tone subtest, but the order of the remaining subtests did change to Duration second and Pitch moving from second to third.

Table S2. Means, standard deviations (SD), effect sizes (female[a] minus male[a]) and implied significance levels (superscripts) for all 19 subtests of the TBAC when ONLY WHITE subjects were included.						
(1)	(2)	(3)	(4)	(5)	6	(7)
Subtest	Subtest Name	Mean		Standard Deviation		Effect
Number[b]	and (Units of Measure)[b]	Females	Males	Females	Males	Size (3 - 4)
1	Pitch (ΔF in Hz)	12.63	10.50	9.35	7.34	**0.242[e]**
2	Intensity (ΔI in dB)	0.75	0.77	0.55	0.58	-0.035
3	Duration (ΔT in ms)	28.85	24.59	16.61	13.25	**0.272[f]**
4	Pulse train (ΔT in ms)	10.12	9.03	5.3	5.08	**0.208[e]**
5	Embedded tone (T in ms)	30.00	25.56	12.39	12.86	**0.354[g]**
6	Temporal order tones (T in ms)	55.72	51.00	27.47	25.52	0.175
7	Temp. order syllables (T in ms)	114.75	127.94	47.19	58.17	**-0.260[f]**
8[c]	Syllable identification (% Correct)	0.74	0.75	0.06	0.06	-0.200
9	SAM[d] 8 Hz (mod. depth in dB)	-24.66	-24.57	3.79	3.71	-0.024
10	SAM 20 Hz (dB)	-23.21	-23.90	4.86	5.03	0.140
11	SAM 60 Hz (dB)	-21.35	-21.04	3.54	3.59	-0.085
12	SAM 200 Hz (dB)	-16.93	-17.37	4.03	3.48	0.116
13	Ripple noise (dB)	-5.55	-6.04	3.36	2.92	0.151
14	Gap detection (T in ms)	2.21	2.02	1.22	1.03	0.166
15	Gap discrimination (ΔT in ms)	37.67	34.70	14.2	13.37	**0.213[e]**
16[c]	Syllable (CVC) identif. (S/N)	-7.55	-7.55	1.53	1.51	0.003
17[c]	Word identification (S/N)	-10.60	-10.57	1.47	1.75	-0.020
18[c]	Sentence identification (S/N)	-8.27	-8.22	0.57	0.60	-0.072
19[c]	Environmental sound identif. (S/N)	-13.03	-13.16	1.13	1.04	0.116

[a]Female N = 213 for most subtests; Male N = 93 for most subtests; otherwise 239 or 99, respectively

[b]From Kidd et al. (2007)

[c]Subtests employing single stimulus presentations; all others were 3-interval, 2-alternative forced choice

[d]Sinusoidal amplitude modulation of a noise band

[e]Implied significance, from resampling: $0.05 < p < 0.10$

[f]Implied significance, from resampling: $0.01 < p < 0.05$

[g]Implied significance, from resampling: $0.001 < p < 0.01$

Although the Ns were small for both the Non-White females (N = 27) and the Non-White males (N = 7), the undersampling was prohibitive only for the Non-White males. Again in the spirit of complete reporting, a comparison of the Non-White and White *females* was implemented. The summary statistics for all 19 subtests are shown separately for the two female groups in Table S3. The effect sizes shown in column (7) were calculated with a numerator of Non-White minus White. Of interest in the pattern of results is: (1) The subtests showing the largest *sex* differences (Embedded Tone,

Pitch, and Duration in Tables S1 and S2) exhibited negligible differences between Non-White and White females (Table S3); (2) the two subtests showing the largest differences between Non-White and White females (Word Identification and Sentence Identification, Table S3) exhibited negligible *sex* differences (Tables SI and S2). The small Ns prohibit definitive conclusions, but the different patterns of results might stimulate follow-up research.

Table S3. Means, standard deviations (SD), effect sizes (Non-White[a] minus White[a]) and implied significance levels (superscripts) for all 19 subtests of the TBAC when ONLY FEMALE subjects were included.

(1)	(2)	(3)	(4)	(5)	6	(7)
Subtest	Subtest Name	Mean		Standard Deviation		Effect
Number[b]	and (Units of Measure)[b]	Non-White	White	Non-White	White	Size (3 - 4)
1	Pitch (ΔF in Hz)	13.04	12.63	10.67	9.35	0.043
2	Intensity (ΔI in dB)	0.98	0.75	0.73	0.55	**0.393**[e]
3	Duration (ΔT in ms)	25.58	28.85	11.87	16.61	-0.202
4	Pulse train (ΔT in ms)	11.15	10.12	6.37	5.30	0.189
5	Embedded tone (T in ms)	30.74	30.00	14.99	12.39	0.058
6	Temporal order tones (T in ms)	58.27	55.72	30.18	27.47	0.092
7	Temp. order syllables (T in ms)	130.85	114.75	58.38	47.19	0.331
8[c]	Syllable identification (% Correct)	0.73	0.74	0.06	0.06	-0.105
9	SAM[d] 8 Hz (mod. depth in dB)	-23.89	-24.66	3.93	3.79	0.202
10	SAM 20 Hz (dB)	-22.42	-23.21	4.66	4.86	0.163
11	SAM 60 Hz (dB)	-20.17	-21.35	3.49	3.54	0.332
12	SAM 200 Hz (dB)	-16.1	-16.93	4.22	4.03	0.205
13	Ripple noise (dB)	-4.22	-5.55	3.30	3.36	**0.398**[e]
14	Gap detection (T in ms)	2.14	2.21	1.28	1.22	-0.057
15	Gap discrimination (ΔT in ms)	40.61	37.67	15.50	14.20	0.205
16[c]	Syllable (CVC) identif. (S/N)	-7.51	-7.55	1.49	1.53	0.021
17[c]	Word identification (S/N)	-9.74	-10.60	1.35	1.47	**0.585**[g]
18[c]	Sentence identification (S/N)	-8.02	-8.27	0.32	0.57	**0.438**[f]
19[c]	Environmental sound identif. (S/N)	-12.68	-13.03	1.15	1.13	0.315

[a]Non-White female N = 27 for most subtests; White female N = 213 for most subtests

[b]From Kidd et al. (2007)

[c]Subtests employing single stimulus presentations; all others were 3-interval, 2-alternative forced choice

[d]Sinusoidal amplitude modulation of a noise band

[e]Implied significance, from resampling: $0.05 < p < 0.10$

[f]Implied significance, from resampling: $0.01 < p < 0.05$

[g]Implied significance, from resampling: $0.001 < p < 0.01$

IIIsupp. C. Correlations

In the reports by McFadden et al. (2018a, 2018b, 2021), correlations were calculated for all possible pairs of physiological measures and behavioral tasks. The goals were to identify possible relationships between physiological and psychoacoustical measures and to identify pairs of psychoacoustical tasks that shared underlying mechanisms. The majority of those correlations were weak.

No parallel set of correlations were done for this re-analysis of the Kidd et al. data. The reason is that such correlations would have limited information value. Our re-analysis used estimated stimulus values that necessarily were assigned in a way that would eliminate extreme values and thereby would compress variability and thus the magnitudes of correlations. (Recall that for our re-analysis, all 34 members of a decile group necessarily were assigned the same value of estimated stimulus value.) Correlations calculated from our assigned stimulus values would be unrepresentative of the relationships underlying TBAC's 19 subtests.

Kidd et al. (2007) did conduct factor analysis on their TBAC results. For that analysis, they used arcsine-transformed values of percent correct scores, a measure that did not constrain extreme values (unlike our estimated stimulus values). For a description of the factor structure underlying the TBAC subtests, see Kidd et al. (2007).

IVsupp. SUMMARY AND DISCUSSION

IVsupp. A. General

When the 19 subtests of the TBAC were examined for sex differences, the majority of the effect sizes were negligible and not significant under resampling (see Table SI). Only one subtest exhibited an effect size greater than 0.3 (Embedded Tone), and while resampling did reveal that sex difference to be highly significant, an effect size of 0.3 still is small by conventional standards (Cohen, 1992).

As noted, that Embedded Tone subtest was rather complex compared to common psychoacoustical tasks. In the Embedded Tone task, each observation interval consisted of a sequence of 9 time slots. The first four time slots and the final four time slots always contained 40-ms tones of different pseudorandom frequencies. The middle (fifth) time slot in the sequence contained either silence (the standard) or a tone of yet another frequency (the alternative stimulus). The duration of the middle

(fifth) time slot was adjusted downward from 200 ms to vary the difficulty of the task. The current duration of the middle tone also was used for the duration of the silent middle time slot in the standard stimulus (first interval) and in the alternative stimulus. The frequencies of the nine tones were chosen at random trial-by-trial from a range of 300 Hz to 3000 Hz, and different sequences of frequencies were used across trials (the sequences within a trial were the same). Note, however, that the specific sequence of frequencies was not relevant to a subject's decision; that decision was simply which observation interval was different from the standard (which had no tone in the middle of the nine-slot sequence). Thus, this task measures the ability to detect the presence/absence of a tone in the middle of a tonal sequence having high uncertainty about the frequencies of those tones.

Another behavioral task known to exhibit a substantial sex difference also involves a form of frequency uncertainty, but in a context of simultaneous masking. Neff et al. (1996) used a masker consisting of 10 tones. Observation interval by observation interval, a different, pseudorandom set of masking tones was selected from the range 0.3 to 3.0 kHz. The duration of those masker tones was 200 ms. The signal, also 200 ms, always was a tone of 1.0 kHz. Thus, there was no uncertainty about the signal but high uncertainty about the masking tones. Neff et al. reported that male performance was about 8 dB better than female performance, which corresponded to an effect size of about 0.73. Using higher frequency tones for signal (3.0 kHz) and masker, McFadden et al. (2018a) confirmed the direction and magnitude of the Neff et al. outcome, and obtained an effect size of 0.67.

Because the Embedded Tone subtest and the Neff et al. task both involve (an irrelevant) frequency uncertainty, future investigators of auditory sex differences will want to examine whether, for some reason, males routinely do better than females when uncertainty (relevant or irrelevant) or auditory complexity is high. Note, however, that males also did better than females on the TBAC task called Pitch, which is a traditional frequency-discrimination task using simple tones.

Past research (reviewed by McFadden, 1998, and McFadden et al., 2018a) suggests that females have better hearing sensitivity than males, exhibit less temporary and permanent noise-induced hearing loss than males, have more overshoot than

males (stronger cochlear amplifiers?), and have less two-tone suppression than males. By comparison, males appear to be more sensitive to both interaural time and interaural level differences (McFadden, 1998, Fig. 1), more sensitive to the cubic difference tone generated between some tonal signals and tonal maskers (McFadden et al., 2012, 2018a), and more sensitive in the Neff et al. (1996) simultaneous-masking task. To the latter list now can be added better male performance in the Embedded Tone and Pitch subtests of the TBAC.

For some common physiological measures of the auditory system, it is easy to interpret the known sex differences as females being "better" than males. Females have stronger spontaneous and click-evoked OAEs (perhaps reflecting stronger cochlear amplifiers?) and faster and stronger click-evoked AEPs (McFadden et al., 2021), and that is true from early in life for both OAEs and AEPs (see McFadden et al., 2021). For psychoacoustical measures, however, the "better" sex varies from task to task (as discussed above and summarized by McFadden et al., 2018a). (This is not just chance variation; findings for various tasks have replicated -- see McFadden et al., 2021.) As a glance at Table SI reveals, the "better" sex also varied across the subtests in the TBAC. Until recently, the question of "better" was interesting because it carried the potential to reveal relationships between physiological and psychoacoustical measures: e.g., do stronger cochlear amplifiers correlate with better behavioral hearing sensitivity or better frequency resolution? However, recent reports revealed only weak correlations between common physiological measures and common psychoacoustical tasks (McFadden et al., 2018b, 2021). Intuition and logic suggest that some strong relationships eventually will be found, but . . . not yet. The eventual explanations for this array of physiological and psychoacoustical sex differences surely will contain elements of "simple" hearing mechanisms and cognitive processes.

IVsupp. B. Future investigations

The TBAC did not include any subtests requiring binaural processing; nor did the collection of tests used by McFadden et al. (2018a). While that choice is understandable from a technical point of view, the omission was unfortunate and is

worthy of remediation by future investigators. The reason is that large individual differences, and some sex differences, have been reported in the literature on binaural hearing (see McFadden, 1998; McFadden et al., 2022).

Hafter and Jeffress (1968) reported that there are separate intracranial images associated with the interaural time cue and the interaural intensity cue, and that people differ in their reliance on those two cues -- individual listeners are biased toward one or the other cue. McFadden et al. (1973) found a similar dichotomy using a stimulus that permitted controlled combinations of interaural level differences and interaural time differences; individual listeners were more sensitive to one of the two cues, and sometimes considerably so.

Langford (experiment summarized in McFadden, 1998) examined how these individual differences cluster to produce sex differences. He measured sensitivity to interaural level differences and to interaural time differences using noise bands and a forced-choice (oddity) task. His 24 male listeners were noticeably more sensitive to both cues than were his 26 female listeners.

When a low-frequency tone is presented to one ear only, and a tone of slightly different frequency is presented to the other ear only, a beat-like experience is heard and lateralized between the ears. These so-called binaural beats have been interesting historically because no beat exists in the physical stimulus; it must arise neurally as a consequence of the slight difference in the neural periodicities in the auditory nerves from the two ears. Tobias (1965) reported a sex difference in binaural beats; his 20 female listeners began to fail to hear binaural beats when the base frequencies (tones) entered the range of about 600 - 800 Hz, whereas his 20 male listeners continued to hear binaural beats until the base frequencies entered the range of about 800 - 1000 Hz, a sex difference worthy of confirmation in more broadly based samples. (McFadden and Pasanen, 1975, demonstrated that binaural beats can be heard all across the auditory spectrum when amplitude-modulated sounds, not pure tones, are used for the base stimuli. Unfortunately, no investigation of sex differences was conducted with those stimuli.)

One final demonstration of sex differences in binaural processing involves more complex stimuli than in the above demonstrations. When two different words are

presented simultaneously to the two ears, and the listener is asked to report both words, most listeners are more accurate at reporting the word presented to the right ear (and this is more pronounced in right-handed listeners). The effect is called the right-ear advantage or REA. While small, the REA has gotten considerable attention over the years from investigators interested in human speech perception, and Kimura and Harshman (1984) reported that their male listeners exhibited a greater REA (more asymmetry) than did their female listeners. A correlated finding is that human male cortices are physically more asymmetric than are those in females, but this may be only a coincidence.

The binaural system appears to be a fertile field for future research on Individual differences, sex differences, and perhaps race differences.

IVsupp. C. Comments

A weakness of this re-analysis of the TBAC data is that estimates of ability had to be assigned to each subject for each subtest using the stimulus values for his/her decile categories (section IIsupp.C). As noted, a likely consequence of this procedure was a reduction in the variability in the two sex groups. When Kidd et al. (2007) fitted sigmoid functions to the group data for each decile, any extreme scores by individual subjects would be out-weighed by the 33 other subjects in that decile. This is relevant because a recurring question of interest in the literature on human sex differences is whether the male distribution of scores exhibits larger variability than the female distribution of scores (topic reviewed by Gray et al., 2016; Summers, 2022). Modest support for "greater male variability" came from AEP, OAE, and behavioral data from a single study (see McFadden et al., 2021), where over 70% of the measures had larger variability for the males. Examination of columns (5) and (6) in Table SI here reveals that the TBAC offers no additional support for this idea; only 6 of the 19 subtests had larger SDs for the males (subtests 2, 5, 7, 10, 17, and 18), and most of those differences were small. The question (which is unanswerable) is whether the variability of one of the two sex groups was disproportionately affected by the procedure used here for assigning stimulus values (section IIsupp.C. above).

As personalized medicine becomes more established as a goal in clinical practice and medical research (Goetz and Schork, 2018), individual differences of various sorts will become of increasing interest. Clusters of individual differences such as sex and race differences also will be of increasing interest. Individual, sex, and/or race differences in the auditory system may prove to have predictive value for prevention or treatment for maladies of the auditory system or for correlated maladies in other systems or organs. The small sizes of the current sex differences do not preclude larger, perhaps clinically relevant, differences for other auditory measures, particularly physiological measures. Furthermore, essentially the entire corpus of current knowledge about human hearing, both physiological and psychoacoustical measures, comes from research done at universities in north America and western Europe and thus is based almost exclusively on White subjects. The existence, and size, of sex (and race) differences in other cultural groups still is unknown, meaning that clinically relevant differences may exist in those groups.

The small sizes of the behavioral differences reported here suggest that they are likely to arise as incidental by-products of responses to evolutionary pressures on characteristics other than audition.

ACKNOWLEDGMENTS

C.S. Watson died prior to the analyses reported here. The original data collection on the TBAC was supported by a research grant awarded to author C. S. W. by the National Institute on Deafness and other Communication Disorders (NIDCD; RO1 DC00250). The additional analyses for sex differences by authors D. M. and E. G.

P. were personally funded. The research of author G. R. K. currently is supported by the National Institute on Deafness and other Communication Disorders (NIDCD; R01 DC013538). The content of this article is solely the responsibility of the authors and does not necessarily represent the official views of the NIDCD or the National Institutes of Health.

REFERENCES

Burns, E. M. (**2017**). “Even-longer-term stability of spontaneous otoacoustic emissions,” J. Acoust. Soc. Am. **142**, 1828–1831.

Burns, E. M., Arehart, K. H., and Campbell, S. L. (**1992**). "Prevalence of spontaneous otoacoustic emissions in neonates," J. Acoust. Soc. Am. **91**, 1571-1575.

Cohen, J. (**1992**). “A power primer,” Psychol. Bull. **112**, 155-159.

Goetz, L. H., and Schork, N. J. (**2018**). "Personalized medicine: Motivation, challenges, and progress," Fertil. Steril. **109**, 952-963.

Gray, H., Lyth, A., McKenna, C., Stothard, S., Tymms, P., and Copping, L. (**2016**). "Sex differences in variability across nations in reading, mathematics and science: A meta-analytic extension of Baye and Monseur," Large-scale Assess Educ. **7**, 1–29.

Hafter, E. R., and Jeffress, L. A. (**1968**). "Two-Image lateralization of tones and clicks," J. Acoust. Soc. Am. **44**, 563-569.

Johnson, D. M., Watson, C. S., and Jensen, J. K. (**1987**). "Individual differences in auditory capabilities," J. Acoust. Soc. Am. **81**, 427-438.

Kidd, G. R., Watson, C. S., and Gygi, B. (**2007**). "Individual differences in auditory abilities," J. Acoust. Soc. Am. **122**, 418-435.

Kidd, G. R., Watson, C. S., and Gygi, B. (**2023**). Individual differences in auditory abilities: The Expanded TBAC dataset. https://doi.org/10.5967/wv6d-n347.

Kimura, D., and Harshman, R. A. (**1984**). "Sex differences in brain organization for verbal and non-verbal functions," Progress Brain Res. **61**, 423-441.

Krizman J, Bonacina S, and Kraus N. (**2019**). "Sex differences in subcortical auditory processing emerge across development," Hear. Res. **380**, 166–174. https://doi.org/10.1016/j.heares.2019.07.002 PMID: 31306931

McFadden, D. (**1998**). "Sex differences in the auditory system," Devel. Neuropsych. **14**, 261-298.

McFadden, D. (**2002**). "Masculinization effects in the auditory system," Arch. Sex. Behav. **31**, 93-105.

McFadden, D. (**2008**). "What do sex, twins, spotted hyenas, ADHD, and sexual orientation have in common?" Perspect. Psychol. Sci. **3**, 309-323.
McFadden, D. (**2009**). "Masculinization of the mammalian cochlea," Hear. Res. **252**, 37-48.
McFadden, D. (**2011**). "Sexual orientation and the auditory system," Front. Neuroendocrinol. **32**, 201-213.
McFadden, D. and Champlin, C. A. (**2000**). "Comparison of auditory evoked potentials in heterosexual, homosexual, and bisexual males and females," J. Assoc. Res. Otolaryngol. **1**, 89-99.
McFadden, D., and Pasanen, E. G. (**1998**). "Comparison of the auditory systems of heterosexuals and homosexuals: Click-evoked otoacoustic emissions," Proc. Natl. Acad. Sci. USA. **95**, 2709-2713.
McFadden, D., and Pasanen, E. G. (**1999**). "Spontaneous otoacoustic emissions in heterosexuals, homosexuals, and bisexuals," J. Acoust. Soc. Am. **105**, 2403-2413.
McFadden, D., and Pasanen, E. G. (1975). "Binaural beats at high frequencies," Science **190**, 394-396.
McFadden, D., Champlin, C. A., Pho, M. H., Pasanen, E. G., Maloney, M. M., and Leshikar, E. M. (**2021**). "Auditory evoked potentials: Differences by sex, race, and menstrual cycle and correlations with common psychoacoustical tasks," PLOS ONE, **16**: e0251363. https://doi.org/10.1371/journal. pone.0251363.
McFadden, D., Jeffress, L. A., and Russell, W. E. (**1973**). "Individual differences in sensitivity to interaural differences in time and level," Percept. Motor Skills, **37**, 755-761.
McFadden, D., Pasanen, E. G., Kidd, G. R., and Gygi, B. (**2024****). "Modest sex differences in the Test of Basic Auditory Capabilities (TBAC)," Frontiers of Psychology, **, ** - **. in press.
McFadden, D., Pasanen, E. G., Leshikar, E. M., Hsieh, M. D., and Maloney, M. M. (**2012**). "Comparing behavioral and physiological measures of combination tones: Sex and race differences," J. Acoust. Soc. Am. **132**, 968-983.

McFadden, D., Pasanen, E. G., Maloney, M. M., Leshikar, E. M., and Pho, M. H. (**2018a**). "Differences in common psychoacoustical tasks by sex, menstrual cycle, and race," J. Acoust. Soc. Am. **143**, 2338-2354.

McFadden, D., Pasanen, E. G., Maloney, M. M., Leshikar, E. M., and Pho, M. H. (**2018b**). "Correlations between otoacoustic emissions and performance in common psychoacoustical tasks," J. Acoust. Soc. Am., **143**, 2355-2367.

Neff, D. L., Kessler, C. J., and Dethlefs, T. M. (**1996**). “Sex differences in simultaneous masking with random-frequency maskers,” J. Acoust. Soc. Am. **100**, 2547–2550.

Nosek, B. A., Alter, G., Banks, G. C., Borsboom, D. et al. (**2015**). "Promoting an open research culture," Science, **348**, 1422-1425.

Open Science Collaboration. (**2015**). “Estimating the reproducibility of psychological science,” Science **349**, 943, aac4716. DOI: 10.1126/science.aac4716.

Rammsayer, T. H., and Troche, S. J. (**2012**). “On sex-related differences in auditory and visual sensory functioning,” Arch. Sex. Behav. **41**, 583–590.

Strickland, E. A., Burns, E. M., and Tubis, A. (**1985**). “Incidence of spontaneous otoacoustic emissions in children and infants,” J. Acoust. Soc. Am. **78**, 931–935.

Summers, V. (**2022**). "Sex differences in number of X chromosomes and X-chromosome inactivation in females promote greater variability in hearing among males," Biol. Sex Diffs. **13,** 49-63. DOI: 10.1186/s13293-022-00457-9.

Surprenant, A. M., and Watson, C. S. (**2002**). "Individual differences in the processing of speech and nonspeech sounds by nornal-hearing listeners," J. Acoust. Soc. Am. **110**, 2085-2095.

Tobias, J. V. (**1965**). "Consistency of sex differences in binaural-beat perception," Internatl. Audiolog. **4**, 179-182.

Watson, C. S., Kelly, W. J., and Wroton, H. W. (**1976**). "Factors in the discrimination of tonal patterns. II. Selective attention and learning under various levels of stimulus uncertainty," J. Acoust. Soc. Am. **60**, 1176-1186.

Watson, C. S., Jensen, J. K., Foyle, D. C., Leek, M. R., and Goldgar, D. E. (**1982a**). "Performance of 146 normal adult listeners on a battery of auditory discrimination tasks," J. Acoust. Soc. Am. **71**, S73.

Watson, C. S., Johnson, D. M., Lehman, J. R., Kelly, W. J., and Jensen, J. K. (**1982b**). "An auditory discrimination test battery," J. Acoust. Soc. Am. **71**, S73.

Watson, C. S., Qui, W. W., Chamberlain, M., and Li, X. (**1996**). "Auditory and visual speech perception: Confirmation of a modality-independent source jof individual differences in speech recognition," J. Acoust. Soc. Am. **100**, 1153-1162.
